# Motion-Based Acuity Task: Full Visual Field Measurement of Shape and Motion Perception

**DOI:** 10.1167/tvst.10.1.9

**Published:** 2021-01-06

**Authors:** Anna Kozak, Michał Wieteska, Marco Ninghetto, Kamil Szulborski, Tomasz Gałecki, Jacek Szaflik, Kalina Burnat

**Affiliations:** 1 Nencki Institute of Experimental Biology, PAS, Warsaw, Poland; 2 Institute of Radioelectronics and Multimedia Technology, Warsaw University of Technology, Warsaw, Poland; 3 Department of Ophthalmology, Medical University of Warsaw, Warsaw, Poland

**Keywords:** Stargardt disease, retinitis pigmentosa, peripheral visual field, central visual field, motion defined shapes discrimination

## Abstract

**Purpose:**

Damage of retinal representation of the visual field affects its local features and the spared, unaffected parts. Measurements of visual deficiencies in ophthalmological patients are separated for central (shape) or peripheral (motion and space perception) properties, and acuity tasks rely on stationary stimuli. We explored the benefit of measuring shape and motion perception simultaneously using a new motion-based acuity task.

**Methods:**

Eight healthy control subjects, three patients with retinitis pigmentosa (RP; tunnel vision), and 2 patients with Stargardt disease (STGD) juvenile macular degeneration were included. To model the peripheral loss, we narrowed the visual field in controls to 10 degrees. Negative and positive contrast of motion signals were tested in random-dot kinematograms (RDKs), where shapes were separated from the background by the motion of dots based on coherence, direction, or velocity. The task was to distinguish a circle from an ellipse. The difficulty of the task increased as ellipse became more circular until reaching the acuity limit.

**Results:**

High velocity, negative contrast was more difficult for all, and for patients with STGD, it was too difficult to participate. A slower velocity improved acuity for all participants.

**Conclusions:**

Proposed acuity testing not only allows for the full assessment of vision but also advances the capability of standard testing with the potential to detect spare visual functions.

**Translational Relevance:**

The motion-based acuity task might be a practical tool for assessing vision loss and revealing undetected, undamaged, or strengthened properties of the injured visual system by standard testing, as suggested here for two patients with STGD and three patients with RP.

## Introduction

The primate retina is highly specialized at the anatomical and functional levels, with the central foveal part mainly devoted to sharp vision of stationary objects and the peripheral part to detecting moving objects and bringing them into foveal vision for further analysis. Damage to the part of the retinal representation of the visual field not only directly impairs its specific processing features but also has an impact on the remaining active part. Accordingly, we recently showed that motion perception, a feature of peripheral processing, is reinforced after binocular central retinal lesions in an animal model of macular degeneration.^1^ Despite many years of investigation, the picture of specific retinal degeneration effects upon human visual processing is not complete. Nevertheless, the perceptual measurements of visual deficiencies in ophthalmological patients are typically separated for either central (shape) or peripheral (motion, space perception) visual properties, and acuity tasks are based solely on the perception of stationary stimuli, as in the Snellen letter chart.

For this reason, we designed a motion-based acuity task that estimates shape and motion perception simultaneously. We used full-screen random-dot kinematograms (RDK), where centrally located shapes were separated from the background by the motion of dots with regard to coherence, direction, or velocity. To check if the contrast in which the motion signal is delivered has an influence on motion-based shape discrimination, we built RDKs in positive contrast, bright dots on a dark background, and in negative contrast, dark dots on a bright background. To date, all the information about positive contrast (ON-type) and negative contrast (OFF-type) processing in the primate visual system derives only from static stimulation of the central part (up to 5 degrees) of the visual field (e.g., see Refs. 2, 3). It is established that the central primate retina is built to optimize and strengthen the negative contrast,^4^ and this domination is preserved at the cortical level.^2^^,^^5^ To our knowledge, there are no direct data about how peripheral processing of motion signals depends on contrast. It is only established that sensitivity to high velocities is specific for peripheral processing,^6^ whereas central motion processing engages slow velocities at higher spatial frequencies presented in positive contrast (ON-type).^7^ To check whether the transient loss of peripheral visual input affects vision in control subjects, we artificially narrowed the visual field to 10 degrees by custom-made goggles. We hypothesized that (1) stimulation of the peripheral visual field will weaken central visual processing and (2) narrowing the visual field will possibly remove the conflict between central and peripheral processing. For preliminary validation of the test, we recruited three patients with retinitis pigmentosa (RP) with progressive peripheral retinal loss and two patients with Stargardt disease (STGD) with central retinal loss to participate in testing. In line with the well-accepted specialization of central and peripheral visual processing, in STGD, central photoreceptor degeneration leads to visual acuity loss and deficits in color vision^8^ (for a review, see Ref. 9). In RP, the progression of photoreceptor degeneration from the periphery to the center of the visual field (for a review, see Ref. 10) leads to motion direction discrimination impairment^11^ and to a reduction of saccade movements,^12^ which is correlated with general problems in visual orientation.^13^

We found that motion acuity tasks in negative contrast and in fast motion, which strongly activates visual peripheries, are the most difficult for all participants, and for patients with STGD, they are unmanageable. Importantly, attenuation of visual periphery stimulation, by diminishing the velocity of RDKs, improves acuity thresholds in all tested subjects. The transient loss of peripheral visual input in control subjects did not significantly affect their thresholds.

## Methods

### Subjects

We tested eight control subjects, three patients with RP and 2 patients with STGD (Table). Control subjects were recruited from Nencki Institute employees. Eight normal-sighted controls underwent routine ophthalmological examinations, which included the Snellen acuity test and color vision, Humphrey perimetry, intraocular pressure measurement, and examination of the anterior segment and eye fundus.

**Table.** Participant Descriptions, Snellen Acuity, and Minimal Perceived Difference Between Stationary Shapes

		Acuity Measurements		
Subjects	Age/Sex	RE	LE	Initial Stationary Thresholds in Degrees (Snellen)
C1	45/f	20/40	20/40	0.15 (20/180)
C2	34/m	20/40	20/40	0.06 (20/72)
C3	30/f	20/40	20/40	0.12 (20/144)
C4	28/f	20/40	20/40	0.09 (20/108)
C5	48/f	20/40	20/40	0.08 (20/96)
C6	29/f	20/40	20/40	0.17 (20/204)
C7	20/f	20/40	20/40	0.15 (20/180)
C8	21/f	20/40	20/40	0.15 (20/180)
RP1	40/m	20/48	20/68	0.09 (20/108)
RP2	31/f	20/25	20/25	0.22 (20/264)
RP3	45/m	20/40	20/40	0.68 (20/816)
STGD1	51/f	20/125	20/160	0.18 (20/216)
STGD2	35/m	20/32	20/40	0.12 (20/144)

Patients with RP with tunnel vision presented a central residual visual field limited to a 10 degrees diameter (Humphrey field analyzer, Samodzielny Publiczny Kliniczny Szpital Okulistyczny), and best-corrected visual acuity equal or superior to 20/40 (Early Treatment Diabetic Retinopathy Study [EDTRS]; see the Table for uncorrected acuity). Clinical examination of patients with RP revealed that the optic disc pallor and pigmentary deposits extended throughout the retina and narrowed blood vessels. In full-field flash electroretinography (ERG; RETIscan, Roland Consult, Germany), rod responses were severely diminished, more than the cone responses, as in rod-cone dystrophy. Multifocal ERG (mfERG) was abnormal.

Patients with STGD had central scotoma, 10 to 20 degrees, without foveal sparing, and best-corrected visual acuity equal or superior to 20/40 (see the Table for uncorrected acuity). Clinical examinations revealed a “bull's eye” appearance of the macula. In flash ERG (RETIscan, Roland Consult), full-field rod responses were normal, and full-field cone responses were either normal or slightly reduced. Multifocal ERG (mfERG) showed decreased responses in central rings, suggesting an abnormal function of the macula. Optical coherence tomography (OCT; Cirrus HD-OT Spectral Domain Technology, Zeiss, Germany) showed a decreased thickness of the retina, most notably in the foveola (Samodzielny Publiczny Kliniczny Szpital Okulistyczny). Patients STGD1 and STGD2 underwent genetic screening and had mutations in the ABCA4 locus, which is typical for monogenic retinal dystrophies in the Central European population.^14^

All methods were performed in accordance with the relevant guidelines and regulations. All procedures were approved by the Ethical Committee, WUM (KB/157/2017, granted to Professor Jacek Szaflik, director of Samodzielnego Publicznego Klinicznego Szpitala Okulistycznego [SPKSO]).

### Stimuli

We used discrimination of Efron shapes, a circle and an ellipse, matched for surface. We previously used similar stimuli to describe the developmental visual deficits in an animal model of congenital cataract.^15^ The positive stimulus was a circle, and the negative stimulus was a vertically oriented ellipse. The task in all conditions was to choose a circle over an ellipse.

**Figure 1.**
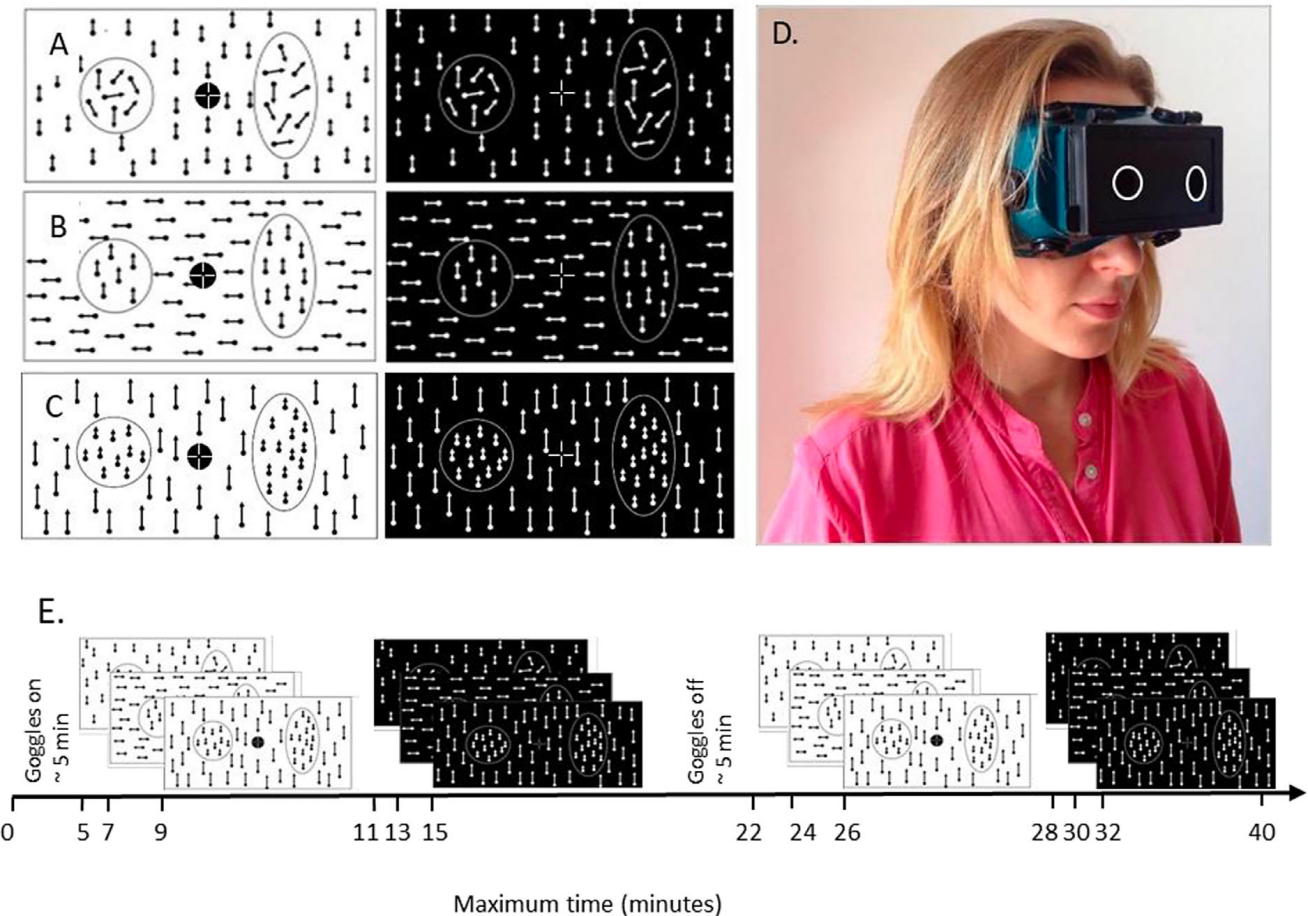
Stimuli and procedure. (**A–C**) Motion-based acuity tasks all shown at the easiest level. Stimuli are defined by motion cues: **A** Coherence (Supplementary Film S1 pos and neg). **B** Direction (Supplementary Film S2 pos and neg). **C** Velocity (Supplementary Film S3 pos and neg). **D** Control subject performing the task with goggles, narrowing the visual field to 10 degrees. **E.** Timeline of the procedure for control subjects. Stimuli were first presented with goggles to model the restricted tunnel vision as in RP and then without goggles. In RP and STGD, the patient's procedure was presented only in an unrestricted visual field.

In the motion-based acuity tasks, circles and ellipses were composed of identical RDKs in either negative or positive contrast. They were separated from background RDK by the motion of dots, by coherence, direction, or velocity:

1. Coherence: S+ and S‒ consisted of dots moving randomly with a velocity of 10 degrees per second. The background was built of dots moving coherently upward with the same velocity as in S+/S‒ (Fig. 1A, Supplementary Film S1 neg and pos).
2. Direction: S+ and S‒ consisted of dots moving coherently upward with a velocity of 10 degrees per second. The background consisted of dots moving coherently leftward with the same velocity as in S+/S‒ (Fig. 1B, Supplementary Film S2 neg and pos).
3. Velocity: We performed three conditions in this task. S+ and S‒ and the background consisted of dots moving coherently upward and dots within S+ and S‒ always moved slower than the background dots: a. 10 degrees per second versus 20 degrees per second; b. 5 degrees per second versus 10 degrees per second; and c. 1 degree per second versus 2 degrees per second (Fig. 1C, Supplementary Film S3 neg and pos).

In all conditions, S+ and S‒ were displayed simultaneously within the central 10 degrees field of view on the RDK background covering 55 degrees. The diameter of the positive stimuli (circle, S+) was 100 pixels (2.88 degrees). The distance between the centers of S+ and S‒ was 220 pixels (6.34 degrees). The fixation point (diameter of 20 pixels, 0.58 degrees) was displayed in the center of the screen according to the guidelines introduced by Thaler.^16^ In the RDKs, every dot's side occupied 0.115 degrees. The dot density was constant for the background and stimuli layers and equal to 11.386 dots/sr (3.47 dots/°^2^). The dots’ velocity was kept constant.

The coherence parameter specified the percentage of dots moving in the same direction. Each dot was assigned a time-dependent displacement vector defined in ℝ^2^, D_cd_ for dots moving coherently, and D_ncd_ for dots moving noncoherently:

$$\begin{array}{ccc} & & \mathrm{if}\;n,\overset{ˇ}{C}§cN:\\ & & \begin{array}{c} ↼\;\;\;-\\ D_{cd} \end{array}\left(t\right)=\left[vt\sin{(}^{''}\;\;\;o\;\bar{I}),vt\cos{(}^{''}\;\;\;o\;\bar{I})\right]\\ & & \mathrm{if}\;n>cN:\\ & & \begin{array}{c} ↼\;\;\;-\;\;-\\ D_{ncd} \end{array}\left(t\right)=\left[vt\sin{(}^{''}\;\;\;o\leq ),vt\cos{(}^{''}\;\;\;o\leq )\right], \end{array}$$

where N – total number of dots, n – dot's identification number, ranging from 1 to N, c – coherence parameter; defines the percentage of dots moving coherently, v – velocity of dots on a given layer, t – time since the beginning of the trial, α – direction of the dot's movement, constant for all coherent dots, and β - direction of the dot's movement, random for each noncoherent dot.

Once a dot left its space of movement, it re-emerged in a random location on the opposing side of the movement area. To preserve an equal distribution of dots inside the display area, the dots moved in a rectangular area larger than the display area.

Stimuli were displayed on an LCD monitor (Iiyama G-Master GE2788HS, 27 inch diagonal, 1920 × 1080 resolution, 60 Hz refresh rate). The screen was split vertically into two halves with each of the stimuli (S+ and S‒) occupying one side of the screen, left or right. The background brightness was constant throughout the whole surface of the display area and equaled 0.152 cd/m^2^ for a dark background and 246 cd/m^2^ for a bright background (measured with a Tektronix J17 photometer). We tested two brightness contrast conditions: (1) negative contrast with dark dots on a bright background (Supplementary Films S1–S3 neg) or (2) positive contrast with bright dots on a dark background (see Figs. 1A–C, left and right panels, respectively; Supplementary Films S1–S3 pos).

The aspect ratio of the ellipse dimensions, depending on the subject answers, could vary from 0.25 to approximately 1, with 1 meaning identical to a circle. The aspect ratio was defined as the ratio between the width (W) and the height (H) of the ellipse:

$$\begin{array}{ccc} & & \text{aspect}\;\text{ratio}=\frac{W}{H}150:\text{was}\;\text{adapted}\;\text{to}\;\text{the}\;\text{subject's}\\ & & \text{performance} \end{array}$$

The difficulty of the task depended on the subject's performance following the adaptive staircase procedure. After a correct response, the subsequent aspect ratio value is calculated according to the formula below (increasing the difficulty):

$$\begin{array}{c} AR_{\mathrm{new}}=\left(H+\left(\mathrm{sqrt}(AR_{\mathrm{old}}\right)-H\right)/\left(1+F\right){)}^{2}, \end{array}$$

after an incorrect response, the formula is as follows (decreasing the difficulty):

$$\begin{array}{c} AR_{\mathrm{new}}=(H+(\mathrm{sqrt}(AR_{\mathrm{old}})-H)(1+{F))}^{2}, \end{array}$$

where:

$$\begin{array}{c} \begin{array}{c} \begin{array}{c} AR_{\mathrm{new}}-\mathrm{new}\;\mathrm{value}\;\mathrm{of}\;\mathrm{the}\;\mathrm{aspect}\;\mathrm{ratio}\;\mathrm{for}\;\mathrm{the}\;\mathrm{ellipse},\\ AR_{\mathrm{old}}-\mathrm{previous}\;\mathrm{value}\;\mathrm{of}\;\mathrm{the}\;\mathrm{aspect}\;\mathrm{ratio}\;\mathrm{for}\;\mathrm{the}\;\mathrm{ellipse},\\ H-\mathrm{the}\;\mathrm{value}\;\mathrm{of}\;\mathrm{the}\;\mathrm{aspect}\;\mathrm{ratio}\;\mathrm{at}\;\mathrm{the}\;\mathrm{highest}\;\mathrm{difficulty}\;\mathrm{of}\;\mathrm{the}\;\mathrm{task},\\ F=0.3,\;\mathrm{determining}\;\mathrm{the}\;\mathrm{pace}\;\mathrm{of}\;\mathrm{changes}\;\mathrm{in}\;\mathrm{the}\;\mathrm{aspect}\;\mathrm{ratio}. \end{array} \end{array} \end{array}$$

The thresholds were calculated based on the aspect ratio of the surface dimensions of the matched circles versus ellipses using the mean calculated from the last four reversals. Reversal means that each time the subject made a mistake by choosing an ellipse over a circle, the task became easier and the difference between the circle and ellipse became more pronounced. Once a threshold was established, we used it to calculate the height of a corresponding ellipse.

$$\begin{array}{c} \mathrm{Ellips}e_{\mathrm{Height}}=\mathrm{Circl}e_{\mathrm{Diameter}/\mathrm{sqrt}}\left(\mathrm{Ellips}e_{\mathrm{AR}}\right) \end{array}$$

**Figure 2.**
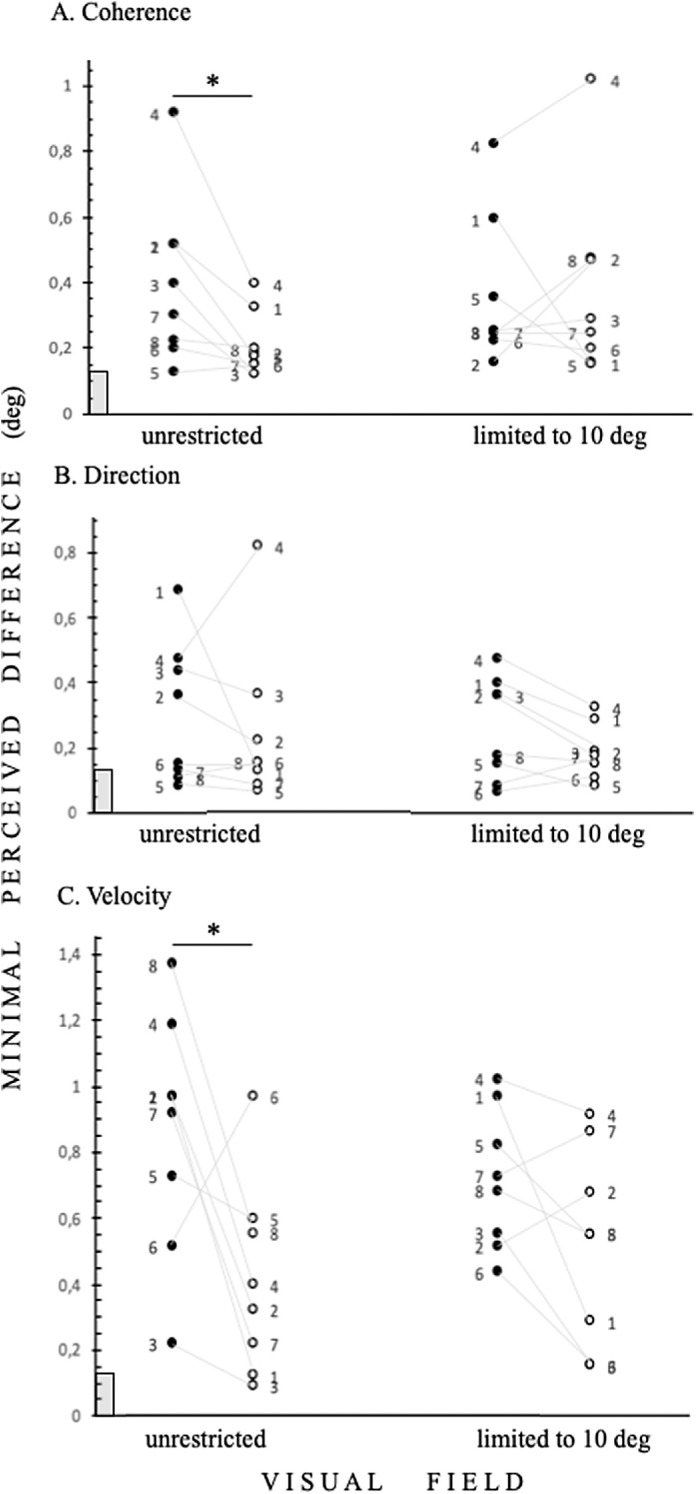
The individual thresholds for minimal perceived differences between circle and ellipse dimensions in visual degrees in control subjects. (**A**) Coherence-based acuity task (see Fig. 1A). (**B**) Direction-based acuity task (see Fig. 1B). Velocity of RDKS 10 degrees per second. Left data column tasks were performed in unrestricted visual fields, and right data columns correspond to tasks performed with limited 10 degrees visual fields by goggles (see Fig. 1D). *Black symbols* represent negative contrast data, *white symbols* represent positive contrast data (see Fig. 1A–C), numbers denote each participant, and gray lines connect personal data from opposite contrasts. The *gray rectangle* denotes 0.15 degrees = 20/60, as measured by the Snellen letter chart. Asterisks indicate significance: * *P* = 0.0234.

**Figure 3.**
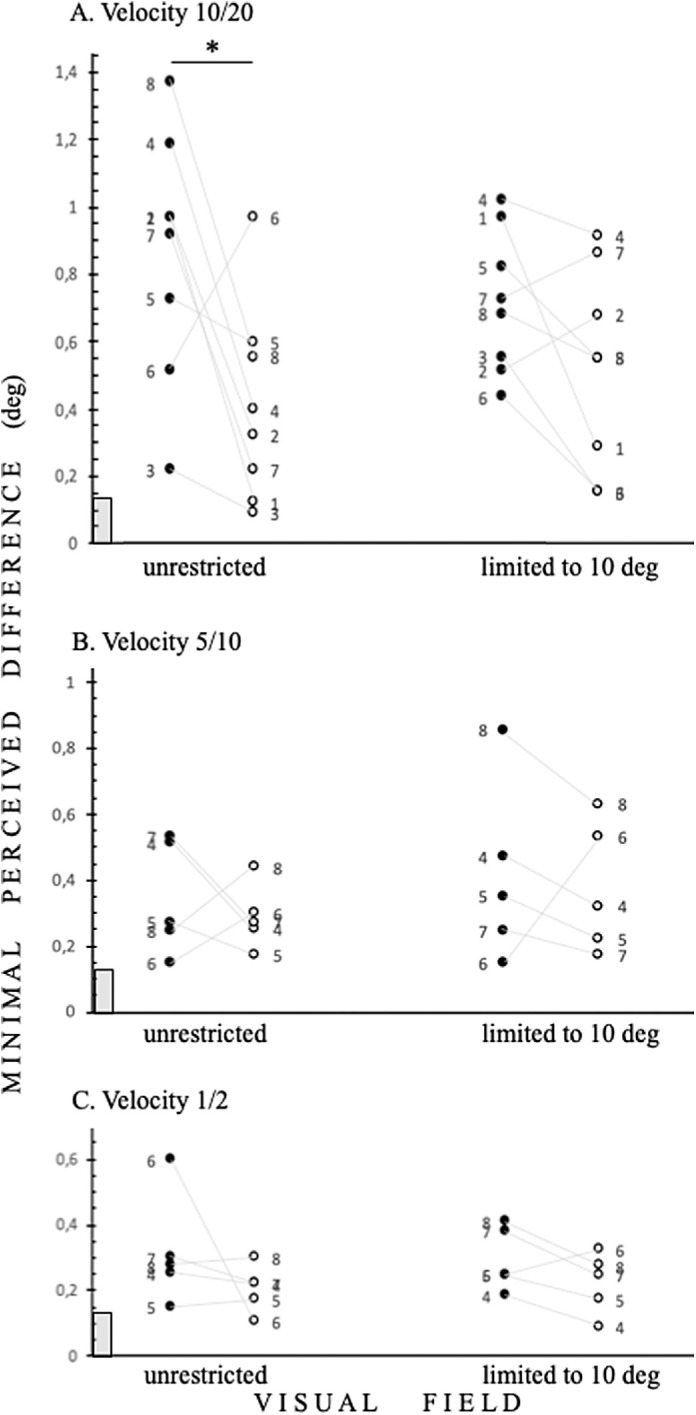
Velocity-based acuity tasks in control subjects. The individual thresholds for minimal perceived differences between circle and ellipse dimensions in visual degrees. (**A**) Velocity-based task, the fastest velocity tested 10/20; (**B**) velocity-based task, 5/10; and (**C**) velocity-based task, the slowest velocity tested 1/2 (degrees per second; shapes versus background). Note that the velocity determines acuity thresholds in negative contrast, as the minimal perceived difference is higher than in positive contrast only at the highest velocity tested, *P = 0.0391. There is no statistical analysis available for **B** and **C**, as we were not able to perform this task on three participants. Other denotations as in Figure 2.

**Figure 4.**
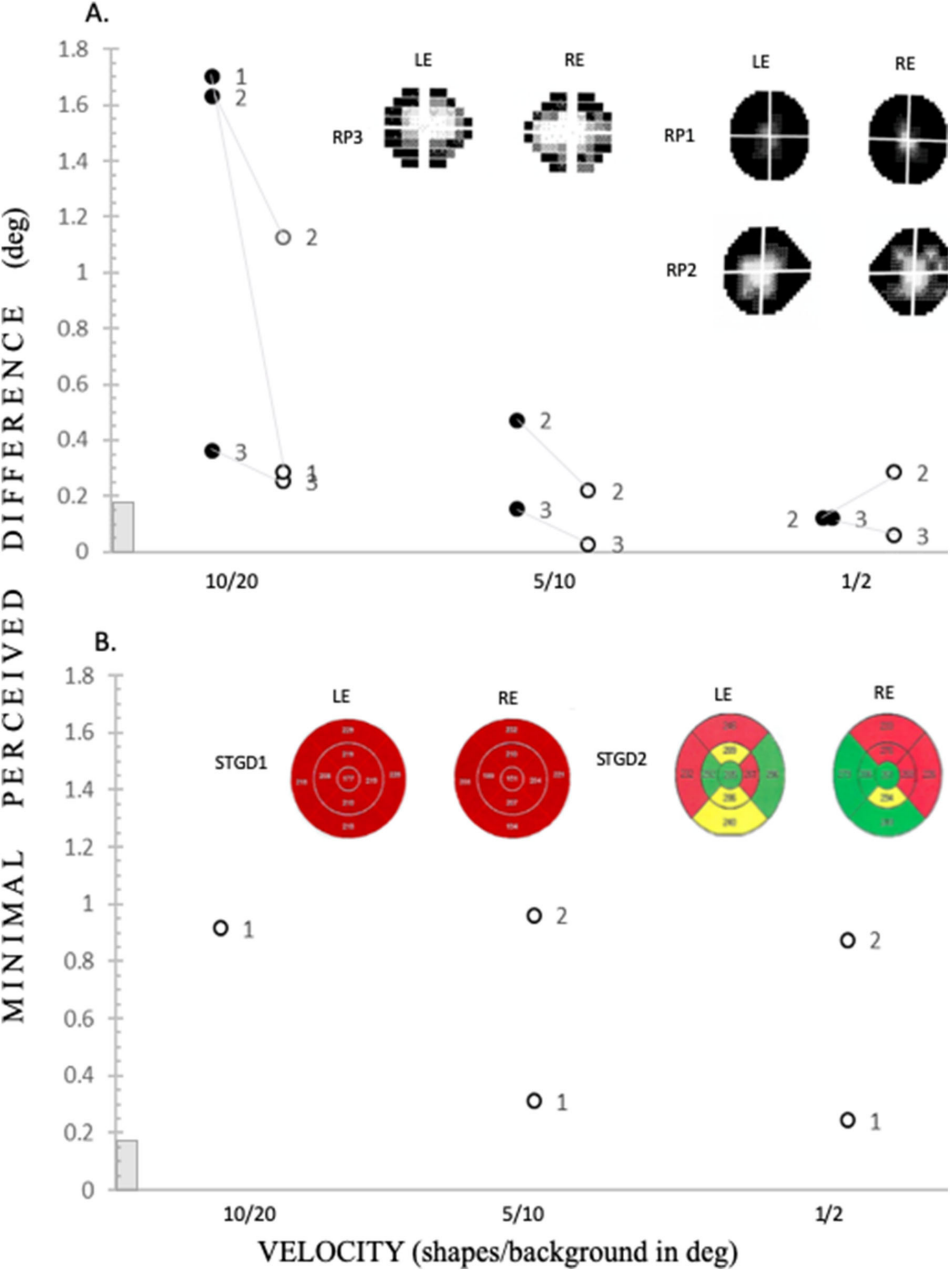
Velocity-based acuity thresholds in patients with RP and patients with STGD performed in unrestricted visual field. The individual acuity thresholds for the minimal perceived difference between circle and ellipse dimensions in visual degrees. Note that decreasing velocity resulted in improved acuity thresholds in both contrasts. (**A**) Patients with RP. Improvement of performance along with a decrease in velocity is observed. Note RP2 has an elevated threshold for the positive contrast with the slowest velocity. RP1 performed only in the fastest 10/20 degrees per second velocity-based task. The grayscale of the Humphrey field analysis (30-2) for each patient is shown. (**B**) Patients with STGD. Note that both patients refused to work in negative contrast. In positive contrast, STGD1 performed at slower velocities, but no improvement in acuity along with a decrease in velocity was observed. STGD2 showed improved performance with decreasing velocity. The velocity of the task is presented in order of testing from fastest to slowest: 10/20, 5/10, 1/2 (velocity within shapes versus in the background degrees per second). The OCT results for both patients are shown: STGD1 all sectors < 1%, STGD2, *yellow* 1 to 5%, *green* > 5%, and *red* < 1%. Other denotations as in Figure 2.

The difference between ellipse height and circle diameter was then expressed in angular degrees, as shown in Figures 2 to 4. Within the Results and Discussion sections, we refer to this value as the threshold.

### Procedure

At the beginning of testing, all participants were familiarized with the procedure and gave written consent to participate in the experiment. Subjects were seated in a dimmed room on a chair at a viewing distance of 57 cm from the screen, with their head located on a chinrest. The stability of the fixation was controlled by an observer using a setup with a camera and separate screen, on which eye and head movements were shown live. Head movements were minimized by the chinrest. In four control subjects, the whole procedure was performed under eye tracker control (EyeLink 1000, SR Research Ltd., Ontario, Canada). Responses were recorded using a keyboard (the left arrow button for the left position of S+ and the right arrow button for the right position of S+). Each subsequent trial started after the subject responded to the previous trial; therefore, the total reaction times of each subject determined the duration of presentation of stimuli in each trial. The subject had a maximum of 10 seconds to give a response; after that time, the procedure continued, and the lack of answers was reported and excluded from the threshold calculation. The presentation of each task lasted for 1 to 2 minutes, which depended on the level of subject performance. Altogether, the whole procedure lasted approximately 40 minutes due to double testing, first with goggles narrowing the visual field and second without (see Fig. 1E).

The initial level of difficulty for the motion-defined tasks was determined using a simple stationary shape discrimination task. Participants were asked to discriminate identical shapes as in the following motion tasks, but stationary plain gray was presented on a plain bright background (53 cd/m^2^ versus 246 cd/m^2^). Then, we measured the distance between eyes in control participants. After adjusting the goggles (Fig. 1D), motion-based acuity tasks were tested, first in the negative contrast and then repeated in the same order in the positive contrast. The whole procedure was repeated after the removal of goggles (unrestricted visual field condition). Figure 1E summarizes the sequence of motion-based acuity task presentations. Three patients with RP and 2 patients with STGD were tested only in unrestricted visual field conditions; therefore, their procedure was shorter than that in control participants (approximately 20 minutes).

## Statistical Analysis

Statistical analyses were performed for all motion-based acuity tasks in the highest 10/20 velocity tested. To analyze the acuity thresholds presented in visual degrees, as the minimal perceived difference between discriminated circles and ellipses, the two-tailed Wilcoxon sign rank test was used. The same Wilcoxon test was performed to compare the number of trials necessary to reach the threshold, and no significant differences between tasks were found. For response time analysis due to the unequal sample size, Welch's *t*-test was used. For all statistical tests performed, the probability level (α-level was set to 0.05) of *P* = 0.05 was accepted as statistically significant. Only significant results are reported in the Results section. Statistical analyses were performed using GraphPad Prism 8 software.

## Results

Using the adaptive staircase method, we measured acuity thresholds for the minimal perceived difference between circle and ellipse dimensions in a set of motion-based acuity tasks defined by the coherence, direction, or velocity of RDKs.

The thresholds for minimal perceived differences between circle and ellipse dimensions in visual degrees are shown in Figures 2 and 3. The acuity thresholds in the negative contrast coherence-based task tested in the unrestricted visual field condition significantly differed from the same task in positive contrast (*P* = 0.0234; Fig. 2A). We revealed that the high-velocity motion-based acuity task in negative contrast tested with an unrestricted visual field is the most demanding task, which is reflected by significantly higher acuity thresholds. The acuity thresholds reached in the negative contrast velocity-based task tested with the unrestricted condition were significantly higher than those reached in the positive contrast velocity-based task tested with the same unrestricted condition (*P* = 0.0391; Fig. 3A). Importantly, this task was also significantly more difficult from all other tasks tested, except for the positive contrast direction-based task tested with the unrestricted condition. The acuity thresholds reached in the negative contrast velocity-based task tested with the unrestricted condition were significantly higher than (1) the coherence-based task with the unrestricted condition in both contrasts, negative (*P* = 0.0156) and positive (*P* = 0.0078), and with the limited condition for both contrasts, negative (*P* = 0.0156) and positive (*P* = 0.0156); and (2) the direction-based task for the unrestricted visual field condition in negative contrast (*P* = 0.0156) and for the limited visual field with both negative (*P* = 0.0156) and positive (*P* < 0.0078) contrast. We also analyzed the differences in achieved thresholds among the presented contrasts, independent of the viewing condition. Again, thresholds in the negative contrast velocity-based acuity task were significantly higher than the positive contrast velocity-based tasks (*P* = 0.0021) and those from all the other tasks: coherence-based positive (*P* < 0.0001) and negative (*P* = 0.0001) contrast; direction-based tasks in positive (*P* = 0.001) and negative contrast (*P* < 0.0002).

Analysis of response times showed that times in the negative contrast coherence-based task with the unrestricted vision condition were significantly longer than those in the positive contrast condition, reflecting significant differences in acuity thresholds (*P* = 0.039, *t* = 2.079, *df* = 181.674; Fig. 2A). Significantly longer response times were also detected for the negative contrast direction-based task tested with the unrestricted condition that for the positive contrast condition (*P* = 0.024, *t* = 2.271, *df* = 222.654). Importantly, for our hypothesis, when the response times were only segregated by the visual field condition, we found that participants responded significantly faster in the visual field limited to 10 degrees (*P* = 0.012, *t* = 2.503, *df* = 1403.044). Additionally, all negative contrast tasks were performed with longer response times, but only for unrestricted visual fields (*P* = 0.013, *t* = 2.470, *df* = 601.230).

This finding made us check whether experimentally weakening peripheral stimulation by reducing velocity in the velocity task would affect thresholds. As predicted, in control subjects, decreasing velocity resulted in a decline in thresholds (see Figs. 3B and C). Unfortunately, we were not able to perform this task on three participants; therefore, we were not able to perform statistical analysis. A comparison of thresholds between all velocity conditions is presented in Figure 3.

Figure 4 summarizes the preliminary validation of the velocity-based acuity test in patients with visual field impairments. For patient RP, as in the control group, negative contrast in all motion-based tasks was the most difficult, and the patients also improved their performance when tested with lower velocities (see Fig. 4A). For the patients with the most severe visual impairment, RP1 and RP2, the highest velocity task in negative contrast was the most difficult, and the difference between positive contrast was strongly pronounced. Interestingly, the coherence task in negative and positive contrast was equally difficult for patient RP1, who had the most severe peripheral visual field loss (acuity thresholds in visual degrees for RP1, RP2, and RP3 for negative and positive contrast, respectively: coherence: 0.86, 0.86; 0.70, 0.80; and 0.83, 0.95, direction: 0.84, 0.93; 0.62, 0.87; and 0.95, 0.97).

For patients with STGD, we were not able to measure thresholds with negative contrast, and patient STGD2 refused to work on the highest 10/20 velocity task with positive contrast (only results from velocity-based acuity tasks are shown in Fig. 4C; acuity thresholds in visual degrees for STGD1 and STGD2, respectively: coherence: 0.29, 0.55; direction: 0.36, 0.29; velocity: 0.91, STGD2 refused). The patient STGD1 with the most severe central visual field impairment was able to perform at lower velocities, although his or her performance did not improve with decreasing velocity (see Fig. 4C). The patient STGD2 did improve his or her performance at lower velocities (see Fig. 4C).

## Discussion

The presented motion-based acuity tasks detect the interference between central and peripheral perception. In healthy control subjects, we show that, irrespective of the extent of visual field stimulation, central motion-based shape processing is disturbed by a high velocity motion-in-negative contrast signal (see Fig. 3A). We know that such a signal strongly activates cortical representation of the peripheral visual field^6^ (for a review, see Ref. 17). Therefore, we conclude that parallel strong activation of the visual field peripheries leads to impaired central processing. We confirmed this finding by reducing the velocity of RDKs by twofold, as it is accepted that peripheral vision processing is strengthened by increasing stimulus size and/or velocity^6^ (for a review, see Ref. 17). Indeed, all tested participants improved their motion-based acuity thresholds compared with the fasted velocity tested (see Fig. 3). The highest significant thresholds were achieved with negative contrast in the unrestricted visual field condition in coherence-based (see Fig. 2A) and highest velocity-based acuity thresholds (see Fig. 3A). This finding is in line with recent results showing that when the central 5 degrees of the visual field is stimulated, subjects more quickly detect moving stimuli in positive contrast than in negative contrast.^7^

Participants responded significantly faster in tasks presented in the visual field limited to 10 degrees. However, to our surprise, we did not find significant differences in acuity thresholds between limited and unrestricted visual fields in control subjects. It is possible that transient, artificial removal of peripheral stimulation by googles is not sufficient to remove well-established components during ontogenetic development facilitation of central visual processing. However, irreversible long-lasting interventions lead to alterations in the balance of central/peripheral visual processing. Earlier, we showed delayed maturation of the cortical representation of the peripheral visual field in an animal model of congenital cataract.^18^ This delay was linked with the processing of negative contrast motion signals (OFF-type) at the retinal and psychophysical levels.^19^^,^^20^ Interestingly, negative-contrast signal domination can also be strengthened in patients with amblyopia tested with static gratings.^3^

This finding is important for reassuring comfort of patients with retinal impairments, as they are stimulated in the daily life by negative contrasts, which are more common in natural images^21^ (for a review, see Ref. 17). Dark visual signals are linked with strong emotions, may be considered an evolutionary residue of the dark predator silhouette, and dominate cortical activity (for a review, see Ref. 22). Low vision patients may be more disturbed by negative contrast stimulation because dark signal domination in natural scenes can be strengthened even more by artificial blurring of the visual scenes.^2^ Indeed, we show that patients with STGD find motion stimulation carried by negative contrast so difficult that they refused to participate. This observation is not surprising if we take into consideration that the central primate retina is built to strengthen the negative contrast and OFF-type visual signal processing,^4^ and this OFF-type domination is preserved at the cortical level.^2^^,^^5^ Most likely, patients with STGD are not able to detect shapes defined by motion in negative contrast, as their central retina is degenerated. Nevertheless, patients with STGD participated when the acuity-from-motion task was based on positive contrast (ON-type stimulation), and they performed even better with slower velocity. This is consistent with recent findings in healthy subjects for restricted stimulation of the central 5 degrees, showing that the ON pathway is involved in slow motion processing, in contrast to the OFF pathway.^7^ It is also well accepted that peripheral vision processing is strengthened by increasing stimulus size and/or velocity^2^^,^^6^ (for a review, see Ref. 17). Therefore, we propose that the visual peripheries of patients with STGD are hypersensitive to high-velocity motion signals in negative contrast, which results in a failure to undertake the centrally driven task. Nevertheless, tasks in positive contrast were possible for them to accomplish, particularly those in slow motion. In fact, all tested subjects performed better with slower velocity, proving that competition between central and peripheral processing plays a strong role even when the active task is placed in the constant central position (Figs. 3 and 4). In the three patients with RP tested, the elevation of motion-based acuity thresholds depended on the extent of peripheral visual field loss (see Fig. 4A). In patients with RP with severe vision loss, photoreceptor degeneration did not result in total ganglion cell death within the peripheral regions of the retina (postmortem retinal ganglion cell count),^23^ suggesting the remaining neural cells of the retina as a possible source for visual restoration. In fact, Luttrull^24^ recently showed improved acuity thresholds in patients with RP after diode micropulse laser monocular treatment applied at the foveal region of the retina. Importantly, for our hypothesis, these improvements were correlated with pattern electroretinography measurements, which were strengthened at 24 degrees of the visual field and not at the location closer to the fovea. This finding suggests that the visual peripheries might hold plastic potential, even in patients with RP with preserved central tunnel vision. In line with this hypothesis for the patients with RP who we tested, negative contrast was more difficult than positive contrast. We hypothesize that the cortical representation of the peripheral retina, although deprived from retinal input in RP, remains functional and might be a potential target of visual rehabilitation strategies (for a review, see Ref. 22). We trust that the proposed motion-based acuity task not only allows full assessment of vision loss but can also uncover potentially undamaged or even strengthened properties of the locally injured visual system that go undetected by standard testing. We suggest that our novel task can be used as an early diagnostic tool at patients’ homes and is useful in scientific research exploring parallel stimulation of central and peripheral visual field at the threshold level.

## Supplementary Material

Supplement 1

Supplement 2

Supplement 3

Supplement 4

Supplement 5

Supplement 6

## Supplementary Material

**Supplementary Video 1.** Supplementary Film 1 pos, coherence-based acuity task in positive contrast.

**Supplementary Video 2.** Supplementary Film 1 neg, coherence-based acuity task in negative contrast.

**Supplementary Video 3.** Supplementary Film 2 pos, direction-based acuity task in positive contrast.

**Supplementary Video 4.** Supplementary Film 2 neg, direction-based acuity task in negative contrast.

**Supplementary Video 5.** Supplementary Flm 3 pos, velocity-based acuity task in positive contrast.

**Supplementary Video 6.** Supplementary Film 3 neg, velocity-based acuity task in negative contrast.

The initial 30 s of the presentation for each task is presented during each film, with the initial ellipse aspect ratio set at 0.43. The participant responded correctly by always choosing the circle.

## References

[1] Burnat K, Hu T-T, Kossut M, Eysel UT, Arckens L Plasticity beyond V1: reinforcement of motion perception upon binocular central retinal lesions in adulthood. *J Neurosci*. 2017; 37(37): 8989–8999. 28821647 10.1523/JNEUROSCI.1231-17.2017 PMC6596799

[2] Jansen M, Jin J, Li X, et al. Cortical balance between ON and OFF visual responses is modulated by the spatial properties of the visual stimulus. *Cereb Cortex*. 2019; 29(1): 336–355. 30321290 10.1093/cercor/bhy221 PMC6294412

[3] Pons C, Jin J, Mazade R et al. Amblyopia affects the ON visual pathway more than the OFF. *J Neurosci*. 2019; 39(32): 6276–6290. 31189574 10.1523/JNEUROSCI.3215-18.2019 PMC6687897

[4] Ahmad KM, Klug K, Herr S, Sterling P, Schein S Cell density ratios in a foveal patch in macaque retina. *Vis Neurosci*. 2003; 20(2): 189–209. 12916740 10.1017/s0952523803202091

[5] Jin JZ, Weng C, Yeh C et al. ON and OFF domains of geniculate afferents in cat primary visual cortex. *Nat Neurosci*. 2008; 11(1): 88–94. 18084287 10.1038/nn2029 PMC2556869

[6] Orban G, Kennedy H, Bullier J Velocity sensitivity and direction selectivity of neurons in areas V1 and V2 of the monkey: influence of eccentricity. *J Neurophysiol*. 1986; 56(2): 462–80. 3760931 10.1152/jn.1986.56.2.462

[7] Luo-Li G, Mazade R, Zaidi Q, Alonso J-M, Freeman AW Motion changes response balance between ON and OFF visual pathways. *Commun Biol*. 2018; 1: 60. 30271942 10.1038/s42003-018-0066-y PMC6123681

[8] Kong X, Strauss RW, Michaelides M, et al. Visual acuity loss and associated risk factors in the retrospective progression of Stargardt disease study. *Ophthalmology*. 2016; 123(9): 1887–97. 27378015 10.1016/j.ophtha.2016.05.027

[9] Cheung SH, Legge GE. Functional and cortical adaptations to central vision loss. *Vis Neurosci*. 2005; 22(2): 187–201. 15935111 10.1017/S0952523805222071 PMC1255967

[10] Narayan DS, Wood JP, Chidlow G, Casson RJ A review of the mechanisms of cone degeneration in retinitis pigmentosa. *Acta Ophthalmol*. 2016; 94(8): 748–754. 27350263 10.1111/aos.13141

[11] Turano K, Wang X. Motion thresholds in retinitis pigmentosa. *Invest Ophthalmol Vis Sci*. 1992; 33(8): 2411–2422. 1634338

[12] Luo G, Vargas-Martin Peli E The role of peripheral vision in saccade planning: learning from people with tunnel vision. *J Vis*. 2008; 8(14): 1–8. 10.1167/8.14.25 PMC2629530 19146326

[13] Vargas-Martin F, Peli E. Eye movements of patients with tunnel vision while walking. *Invest Ophthalmol Vis Sci*. 2006; 47(12): 5295–5302. 17122116 10.1167/iovs.05-1043 PMC1752198

[14] Ścieżyńska A, Oziębło D, Ambroziak AM, et al. Next-generation sequencing of ABCA4: high frequency of complex alleles and novel mutations in patients with retinal dystrophies from Central Europe. *Exp Eye Res*. 2016; 145: 93–99. 26593885 10.1016/j.exer.2015.11.011

[15] Burnat K, Stiers P, Arckens L, Vandenbussche E, Zernicki B Global form perception in cats early deprived of pattern vision. *Neuroreport*. 2005; 16(7): 751–754. 15858419 10.1097/00001756-200505120-00019

[16] Thaler L, Schütz A, Goodale M, Gegenfurtner K What is the best fixation target? The effect of target shape on stability of fixational eye movements. *Vision Res*. 2012; 76: 31–42. 23099046 10.1016/j.visres.2012.10.012

[17] Strasburger H, Rentschler I, Jüttner M Peripheral vision and pattern recognition: a review. *J Vis*. 2011; 11(5): 13. 10.1167/11.5.13 PMC11073400 22207654

[18] Laskowska-Macios K., Zapasnik M., Hu T. T., Kossut M., Arckens L., Burnat K. Zif268 mRNA expression patterns reveal a distinct impact of early pattern vision deprivation on the development of primary visual cortical areas in the cat. *Cerebral Cortex*. 2015; 25(10): 3515–3526. 25205660 10.1093/cercor/bhu192 PMC4585500

[19] Burnat K, Gucht EV, Waleszczyk WJ, Kossut M, Arckens L Lack of early pattern stimulation prevents normal development of the alpha (Y) retinal ganglion cell population in the cat. *J Comp Neurol*. 2012; 520(1): 2414–29. 22237852 10.1002/cne.23045

[20] Zapasnik M, Burnat K. Binocular pattern deprivation with delayed onset has impact on motion perception in adulthood. *Neurosci*. 2013; 255: 99–109. 10.1016/j.neuroscience.2013.10.005 24120559

[21] Ratliff CP, Borghuis BG, Kao Y-H, Sterling P, Balasubramanian V Retina is structured to process an excess of darkness in natural scenes. *Proc Natl Acad Sci USA*. 2010; 107(40): 17368–17373. 20855627 10.1073/pnas.1005846107 PMC2951394

[22] Burnat K Are visual peripheries forever young? *Neural Plast*. 2015; 2015: 307929. 25945262 10.1155/2015/307929 PMC4402573

[23] Humayun MS, Prince M, de Juan E Jr, et al. Morphometric analysis of the extramacular retina from postmortem eyes with retinitis pigmentosa. *Invest Ophthalmol Vis Sci*. 1999; 40(1): 143–148. 9888437

[24] Luttrull J. Improved retinal and visual function following panmacular subthreshold diode micropulse laser for retinitis pigmentosa. *Eye*. 2018; 32(6): 1099–1110. 29449615 10.1038/s41433-018-0017-3 PMC5997672

